# Childhood Adversity Accelerates Intended Reproductive Timing in Adolescent Girls without Increasing Interest in Infants

**DOI:** 10.1371/journal.pone.0085013

**Published:** 2014-01-16

**Authors:** Stephanie Clutterbuck, Jean Adams, Daniel Nettle

**Affiliations:** 1 Centre for Behaviour and Evolution, Institute of Neuroscience, Newcastle University, Newcastle upon Tyne, United Kingdom; 2 Institute of Health & Society, Newcastle University, Newcastle upon Tyne, United Kingdom; Durham University, United Kingdom

## Abstract

Women experiencing greater childhood adversity exhibit faster reproductive trajectories. One possible psychological mechanism underlying this phenomenon is an increased interest in infants. Interest in infants is thought to be an adaptation important for successful rearing as it motivates the acquisition of caretaking skills. We investigated the relationships between childhood adversity, intended reproductive timing and interest in infants in a sample of English adolescent girls. Specifically we sought to investigate the relationship between 1) childhood adversity and intended reproductive timing; 2) childhood adversity and interest in infants; and 3) intended reproductive timing and interest in infants. Additionally we explored different methods of measuring interest in infants using self-reported fondness for babies, a forced choice adult versus infant paper-based preference task and a novel computer based attention task using adult and infant stimuli. In total 357 girls aged nine to 14 years participated in the study, which took place in schools. Participants completed the two interest in infants tasks before moving on to a childhood adversity questionnaire. Girls with more childhood adversity reported earlier ideal ages at parenthood. We found some evidence that, contrary to our predictions, girls with less childhood adversity were more interested in infants. There was no relationship between intended reproductive timing and interest in infants. The different measurements for interest in infants were only weakly related, if at all, highlighting the complexity of measuring this construct. Our findings suggest that rather than interest in infants being a mechanism for the effect of childhood adversity on early reproductive timing it might instead be an indicator of future reproductive strategies.

## Introduction

A number of influential evolutionary theories have hypothesized that adversity experienced in childhood accelerates reproductive timing [1]–[4].The adaptive basis of this acceleration is that when prospects are poor, it is adaptive to reproduce early to maximize the chances of producing at least some offspring who will themselves reach reproductive maturity. Empirical research, largely from US and UK populations, supports these theories: areas of high unemployment, poverty and male incarceration have increased rates of teenage pregnancy [5]. What is more, unpredictability within the family and low parental investment via maternal or paternal absence [6]–[8], negative subjective feelings about parental support [9], and frequent residential moves in early childhood are more common in young mothers [7]. The impact of low parental investment on reproductive timing can begin even at the perinatal stage: having a young mother [10], experiencing reduced duration of breastfeeding, being born early for gestational age and of low birth weight [7] are all associated with earlier age at first birth in females. At the macro level, neighbourhoods with shorter life expectancy and increased homicide rates have younger ages at first birth [11], [12]. Additionally reproductive timing mimics neighbourhood gradients of healthy life expectancy in the UK with an average decrease of seven years in age at first birth for those who can expect the fewest healthy years [12]. Even perceptions of neighbourhood safety [13] and personal disadvantage [14] appear to be strongly associated with accelerated reproduction.

The psychological mechanisms facilitating the effects of these antecedents on reproductive timing have received less attention than the social [15] or hormonal mechanisms [16]–[19]. However, one psychological mechanism given some consideration is an increased level of interest in infants in girls who have experienced early adversity [20]. There is evidence in the human and non-human primate literature that interest in infants is an adaptation to acquire sufficient parenting skills for offspring survival and ultimately increase inclusive fitness (for review see [21]). Evidence from non-human primates has found better survival outcomes for infants reared by mothers who showed higher interest in infants prior to reproducing [22]. Interest in infants tends to be higher in females than males, peaking around adolescence and declining with age, mirroring the female reproductively viable years [23]–[27]. Lorenz [28] proposed that it is the characteristic morphology of round face and forehead and large eyes, termed ‘Kindchenschema’ or ‘baby schema’, that makes an infant attractive and motivates nurturing behaviour. Physiological support for the motivational effect of baby schema is evident in the increased activation of the neural areas linked to the brain's reward centre when women view images of infants high in these distinctive features [29]. Increased sensitivity to infantile features is considered an adaptation important for mother-infant bonding and ultimately resource allocation amongst multiple offspring [30].

Although Maestripieri et al. [20] found evidence that interest in infants was associated with early adversity, their measurement of adversity was limited to family structure and the quality of the family environment. In their study, only father absence was associated with their proxy for reproductive timing (menarche), and, independently, with interest in infants. Maestripieri et al. [20] were able to demonstrate a weak direct link between reproductive timing and interest in infants, such that girls with early menarche preferred images of infant stimuli more than girls with later menarche. However, they argued this relationship arose from the strong associations of both early menarcheal timing and interest in infants to father absence. Similar to Maestripieri et al. [20] we propose that if acquisition of mothering skills is important before parenthood then girls experiencing more adversity will be on faster reproductive trajectories and consequently will display increased interest in infants for their age. The aim of our study was to investigate this hypothesis using a larger, more diverse population than Maestripieri et al. [20], and a broader range of childhood adversity variables, encompassing both family-level and neighbourhood-level factors.

Unfortunately, there is no consensus on the best way to measure interest in infants. Methods have included interactions with unknown infants both whilst mothers were [24], [31]–[33] and were not present [23], [34]; measurement of skin conductance, heart rate, facial muscle movements and neural activation when exposed to images of infants [25], [29], [35]; preference for infant stimuli over adult stimuli and desire to view infant stimuli longer, rate them as more attractive, or pay more attention to them [26], [36]–[40]; and reported desire to spend time with infants and interact with them in hypothetical social situations [27].

Thus, we also sought to explore alternative approaches to measuring interest in infants. Because we are measuring similar hypotheses within a similar age group we used the same Preference Task as Maestripieri et al. [20], [27]. However, this forced-choice paper and pencil task is an explicit measure that may introduce social desirability bias. Therefore, we wanted to also measure interest in infants implicitly via attention. Some popular methods for measuring attention include, but are not limited to, visual attention using eye tracking devices [41], orienting paradigms such as dot probe tasks [42] and motivation driven key-press tasks. Some of these methods have been used in relation to measuring interest in infants [39], [40], [43]. However, eye tracking devices were not appropriate for non-laboratory settings such as the schools where we collected data, and reliability of dot-probe tasks in non-clinical participants has been contested [42]. As well we were concerned a key-press task might confuse our participants, particularly those at the younger end of the age range, which could lead to disengagement with the task. We, therefore, developed a novel tool based on the idea that participants who were highly interested in infants would have their attention more easily captured by infant images, as opposed to adult images, during an unrelated task and would later have better memory for those infant images. This tool combined a simple computer based object search task and an unexpected recognition task of infant and adult faces. Finally, our third method of measuring interest in infants was a self-reported fondness for babies questionnaire item.

## Aims

This study had four aims: 1) To explore different methods of measuring interest in infants; 2) To establish whether interest in infants in young females is associated with earlier intended reproductive timing; 3) To investigate whether childhood adversity predicts intended reproductive timing; and 4) To investigate whether childhood adversity predicts interest in infants.

## Methods

### Study Overview

Girls aged nine to14 years were recruited via schools in one local authority area in the North East of England. Information letters and consent forms were sent home to parents inviting their daughter to take part in the study. This was a cross-sectional study completed in schools via self-report questionnaires, and paper-based and computer-based tasks. Information on participant's menarcheal status was also collected. However, menarche was not related to interest in infants or ideal age at parenthood and was only related to two of the childhood adversity measures. As such we felt the variable did not add significantly to the findings of the paper and was not included in the current analysis. The measures gathered in the study are summarised in Table 1.

**Table 1:** Summary table of the study measures.

Interest in Infants	Childhood Adversity	Intended Reproductive Timing
Fondness for Babies	Neighbourhood Deprivation	Ideal Age at Parenthood
1PT: Animal Infant Silhouettes	Residential Instability	
1PT: Human Infant Silhouettes	3Mother Absence	
1PT: Animal Infant Photographs	3Timing of Mother Absence	
1PT: Human Infant Photographs	Father Absence	
2CPTT: Accuracy	4Timing of Father Absence	
2CPTT:Time	Step Father Presence	
	Biological Brothers	
	Biological Sisters	
	Half/Step Brothers	
	Half/Step Sisters	
	Family Support	
	Perceived Neighbourhood Safety and Quality	

^1^ PT refers to Preference Task.

^2^ CPTT refers to Count the Purple Triangles Task.

^3^ Mother Absence and Timing of Mother absence were not used in the analysis because only 5% of the participants had experienced this event.

^4^ This consisted of two categories for Timing of Father Absence: 1) 0 to 5 years, 2) 6 years to 14 years.

### Ethics Statement

Ethical approval for the study was obtained from Newcastle University's Faculty of Medical Sciences Ethics Committee. Written parental consent was required for participation in the study.

### Measures of Interest in Infants

#### Fondness for babies

The questionnaire item ‘How much do you like babies?’ was measured on a scale of 1 (not at all) to 7 (very much).

#### Preference task (PT)

This consisted of a booklet with 20 pairs of adult vs. infant images. The images were grouped as (with Cronbach's α scores, [20]): five animal face silhouettes (α = 0.66), five human face silhouettes (α = 0.85), five animal face photographs (α = 0.75), five human face photographs (α = 0.71). Human photographs included both sexes. Maestripieri et al. [27] included silhouettes because they are void of individual identity and therefore reflect perceptual bias toward infantile features. Participants were instructed to choose the preferred image from each of the pairs. The four outcome measures from this task were total number of infant images chosen as preferred from each of these categories.

#### Count the purple triangles task (CPTT)

This was a computer-based task programmed using E-Prime version 2.0. It consisted of 24 trials with an adult or an infant face presented in the middle of the screen surrounded by purple and blue triangles and purple squares. Participants were instructed to count the number of purple triangles on the screen and enter the number before moving on to the next trial. After completing the trials participants were given an unexpected recognition task where 24 trials of infant or adult face images were presented on the screen one at a time. Half of the images (six infants, six adults) had been presented during the initial counting task. Participants were asked to indicate if they remember seeing the face by pressing the ‘y’ (yes) or the ‘n’ (no) key. The experimental trials were preceded by three practice trials using inanimate objects in place of faces. There were two outcome measures from this task. The first was accuracy at recognising the category of image (i.e. adult or infant). This was calculated using Cohen's κ score of the agreement between the participant's response in the recognition part of the task and actual presence or absence of that image during the counting phase. Participant's ‘adult’ Cohen's κ score was subtracted from their ‘infant’ Cohen's κ score to create the CPTTAcc variable. Thus a score of a positive value indicated better accuracy for recognising infant stimuli. The second outcome measure was the distractibility of the stimuli. For this the average time (in milliseconds) that it took participants to count the purple triangles whilst an adult or infant image was presented on the screen was calculated. Participant's ‘adult’ time was subtracted from their ‘infant’ time to create the CPTTTime variable. Again a positive value indicated more time searching for purple triangles while an infant was on the screen.

### Measures of Childhood Adversity

#### Neighbourhood deprivation

Neighbourhood deprivation was measured as the rank of the Index of Multiple Deprivation score of area of residence, identified using postcode of residence. Index of Multiple Deprivation is a small-area based marker of deprivation calculated using a range of measures in seven domains (income; employment; health and disability; education, skills, and training; barriers to housing and services; crime; and the living environment) and is the UK government's preferred marker of deprivation [44]. All small areas, referred to as Lower Super Output Areas (LSOA), in England and Wales are ranked from 1 (most deprived) to 32,482 (least deprived).

#### Residential instability

Participants reported the number of times they had moved house.

#### Family structure

Parental and step-parental residence in the home was reported along with age, if applicable, at parental separation. In line with Draper and Harpending [4], who proposed that the first five years of life were particularly sensitive to father absence, we created the Timing of Father Absence variable. For consistency we also created the Timing of Mother Absence variable. Step-father presence was derived indirectly, such that those participants who reported father absence as well as step-parent presence in the home were recorded as having a step-father living at the same residence. Total number of biological brothers and sisters as well as half/step brothers and sisters was also reported.

#### Family support

This scale captured the extent to which participants felt their parent(s) cared for their well-being. The scale was modified from the Family Stress Scale [45] as used by Nettle & Cockerill with a scale reliability of α = 0.78 [15]. It included five questions (e.g. ‘My father is always there when I need him’) measured on a seven-point scale (‘1 Strongly Disagree’ to ‘7 Strongly Agree’). Scores were summed for analysis and higher scores indicated stronger feelings of family support.

#### Perceived neighbourhood safety and quality

The perceived neighbourhood safety and quality scale measured feelings of safety and exposure to delinquent behaviours in the neighbourhood (e.g. ‘Most adults in my neighbourhood respect the law’) using eight items from the 18 item Neighbourhood Environment Scale [46]. Participants indicated how true each statement was for them (e.g. ‘Not at all true’, ‘A little true’, ‘Sort of true’, ‘Very true’). Responses were summed, with higher scores indicative of better perception of neighbourhood.

### Measure of Intended Reproductive Timing

Participants were asked to circle ‘Yes’ or ‘No’ to the following questions: ‘Would you like to have children one day?’ then, ‘If you answered yes, How old would you like to be when you have your first child? Participants wrote down their desired age at first birth in the space provided. Although not directly comparable, previous longitudinal research has shown that reproductive intentions stated in late adolescence are strong predictors of subsequent reproductive behaviour [9]

### Procedure

Participants took part in groups of two to four during school hours in a quiet room and were given verbal and written instructions. Participants first completed the PT or the CPTT, with the order of completion counterbalanced. All participants completed the questionnaire after the PT and CPTT were completed. For the CPTT the laptops were positioned such that other participants and the researcher could not see the screen while the participants completed the task.

### Data Analysis

If any answer on the PT or the questionnaire was left blank a mid-point was imputed. If participants put an age range for intended reproductive timing the midpoint was also imputed. However, if multiple ages for intended reproductive timing were given the mean was taken. Univariate and multivariate general linear models (GLM) determined which childhood adversity factors were associated with interest in infants and reproductive timing. Paired t-tests determined infant preference and accuracy during the PT and the CPTT as well as time to complete object search during CPTT. Data was analysed using SPSS version 19 and all tests were two-tailed with a p value of <0.05 regarded as statistically significant.

## Results

### Descriptive Statistics

In total, 357 girls took part in the study. Three girls were omitted from the final analysis because one was older than the cut off age and two had previously taken part in a similar study run by the research team. This left 354 girls whose data were included in the final analysis, though the computer-based data from one further participant was omitted from the relevant analysis as she required the aid of the researcher to complete the task. The distributions of ages in the final samples were as follows: 9 (n = 45), 10 (n = 103), 11 (n = 76), 12 (n = 71), 13 (n = 42), 14 (n = 17).

Descriptive data on demographics, family structure and intended reproductive timing are summarised in Table 2. One quarter of participants resided in the 20% most deprived areas in England and Wales, with a further one quarter living within the 27% most affluent. Over a third of participants (37%) had never moved house with almost half (48%) moving anywhere from one to three times. A further 12% had moved house from four to six times and only 4% had relocated more than six times. Five percent of the participants had experienced mother absence from the home compared to 36% who had experienced father absence. Father absence occurred at age five or younger for 60% of the father absent girls with 40% reporting ages of six or older at the time of the event. One participant stated her father currently lives in the same house as her but that she was five when he stopped living in the same house. Although this seems contradictory it is possible that the participant's father was seperated from her mother sometime during the ages of zero to five years and subsequently reconciled. For the purposes of the analysis this participant was categorised as currently ‘father present’ but with ‘father absence’ between 0 to 5 years’. Of those whose father no longer lived in the same house 38% had a step-father living in the same house. The majority of participants (91%) had one or more biological (80%) or non-biological sibling (35%). In this sample nearly all participants (91%) stated a desire to have children one day.

**Table 2:** Descriptive statistics for the study measures.

			Range	
Study Measures		Mean (St. Dev)	Min	Max
Interest in Infants				
	Fondness for Babies	5.47 (1.70)	1	7
	1PT: Animal Infant Silhouettes	1.82 (1.62)	0	5
	1PT: Human Infant Silhouettes	3.52 (1.22)	0	5
	1PT: Animal Infant Photographs	4.25 (0.89)	1	5
	1PT: Human Infant Photographs	3.77 (1.26)	0	5
	2CPTTAcc	−0.14 (0.35)	−1.40	0.83
	3CPTTTime	71.70 (426.37)	−3299.17	2268.75
9Childhood Adversity				
	Neighbourhood Deprivation 4LSOA	15091.08 (9876.85)	507	31911
	Residential Moves	1.76 (2.28)	0	18
	5Age at Mother Absence	5.66 (4.21)	0	13
	6Age at Father Absence	4.68 (3.95)	0	14
	Biological Brothers	0.68 (0.79)	0	4
	Biological Sisters	0.63 (0.78)	0	4
	Half/Step Brothers	0.36 (0.82)	0	6
	Half/Step Sisters	0.37 (0.72)	0	4
	7Family Support	29.02 (5.48)	10	35
	8Perceived Neighbourhood Safety and Quality	26.82 (4.05)	10	32
Intended Reproductive Timing				
	Ideal Age at Parenthood	24.97 (3.90)	14	36

^1^ PT: Preference Task.

^2^ CPTTAcc: the difference in accuracy of remembering infant versus adult faces during the unexpected recognition part of the Count the Purple Triangles Task. Positive value indicates better accuracy for infants.

^3^ CPTTTime: the difference in time (milliseconds) spent searching for purple triangles when a baby is on the screen compared to when an adult is on the screen during the Count the Purple Triangles Task. Positive value indicates more time spent searching while infants were on the screen.

^4^ LSOA: Lower Super Output Area. It is an Index of Multiple Deprivation ranking small areas in England and Wales on a scale from 1 (most deprived) to 32,482 (least deprived).

^5^ Age at Mother Absence: the age at which mother stopped living in the same residence as participant (n = 17).

^6^ Age at Father Absence: the age at which father stopped living in the same residence as participant (n = 127).

^7^ Family Support: the minimum possible score was 5 and the maximum was 35, higher scores indicate more positive feelings of family support.

^8^ Perceived Neighbourhood Safety and Quality: the minimum possible score was 8 and the maximum was 32, higher scores indicate more positive perceptions of neighbourhood.

^9^ Childhood Adversity: Timing of Father Absence and Step Father Presence are not included in this table because they are categorical variables. They are discussed in the text of the Results section.

### Interest in Infants

Table 2 shows the descriptive statistics for the interest in infants measures. In the PT, participants demonstrated a higher preference for infant images (M = 13.36, SD = 3.07) as compared to adult images (M = 6.53, SD =  2.99) t(353) =  21.30, p = 0.001. They preferred infant photos (M = 8.02, SD = 1.70) more than infant silhouettes (M = 5.34, SD = 2.12), t(353) = 21.96, p = 0.001. Participants showed higher preference for the human infant images (M = 7.29, SD = 1.89) than animal infant images (M = 6.07, SD = 1.93), t(353) = 10.03, p = 0.001. This preference for human infant images was only evident for the silhouettes, with human infants silhouettes (M = 3.52, SD = 1.22) being preferred more than animal infant silhouettes (M = 1.82, SD = 1.62), t = (353) = 16.55, p = 0.001. In contrast animal infant photos were preferred more (M = 4.25, SD = 0.89) than human infant photos (M = 3.77, SD = 1.26), t(353) = 6.62, p = 0.001. However, within the categories of images human infant photos were preferred more than human infant silhouettes, t(353) = 2.99, p = 0.003 and likewise animal infant photos were preferred more than animal infant silhouettes, t(353) = 26.10, p = 0.001.

In the CPTT, participants were more accurate at recognising adult images (M = 0.28, SD = 0.29) than they were at recognising infant images (M = 0.14, SD = 0.27), t(342) = 7.50, p = 0.001. However, time spent searching and counting the purples triangles was longer during the infant stimuli trials (M = 1231.13, SD = 585.99) than the adult stimuli trials (M = 1159.43, SD = 570.85), t(351) =  3.16, p = 0.002.

### Relationships between Measures of Interest in Infants and Intended Reproductive Timing

As seen in Table 3, none of the correlations between interest in infant measures reached traditional cut-offs for moderate or strong effect sizes. Fondness for Babies was weakly but significantly positively correlated with three of the four PT scores (Human Infant Silhouette, Animal Infant Photo, and Human Infant Photo). Scores on the CPTT were not significantly correlated with those from any of the other tasks. Within the CPTT, time spent searching (CPTT Time) was not correlated to participant's accuracy (CPTTAcc). There were no statistically significant correlations between intended reproductive timing and any of the measures of interest in infants.

**Table 3:** Correlation coefficients between measures of interest in infants and intended reproductive timing.

	Fondness for Babies	1PT: Animal Infant Silhouettes	1PT: Human Infant Silhouettes	1PT: Animal Infant Photos	1PT: Human Infant Photos	2CPTTAcc	3CPTTTime
Fondness for Babies							
1PT: Animal Infant Silhouettes	0.08						
1PT: Human Infant Silhouettes	0.15*	0.10					
1PT: Animal Infant Photos	0.11*	0.12*	0.15*				
1PT: Human Infant Photos	0.12*	0.22*	0.17*	0.23			
2CPTTAcc	0.05	−0.04	0.00	0.04	0.02		
3CPTTTime	−0.03	−0.02	−0.02	0.02	0.07	0.05	
Ideal Age at Parenthood	−0.05	−0.03	0.03	−0.04	−0.04	−0.02	0.01

p<0.05.

^1^ PT: Preference Task.

^2^ CPTTAcc: the difference in accuracy of remembering infant versus adult faces during the unexpected recognition part of the Count the Purple Triangles Task.

^3^ CPTTTime: the difference in time (milliseconds) spent searching for purple triangles when a baby is on the screen compared to when an adult is on the screen during the Count the Purple Triangles Task.

### Childhood Adversity and Intended Reproductive Timing

The childhood adversity variables plus age were added into a univariate GLM as predictor variables with intended reproductive timing as the outcome variable (see Table 4). Higher neighbourhood deprivation, more frequent residential relocation, having more half/step brothers, feeling less supported by family and having lower perception of their neighbourhood were associated with a younger ideal age at parenthood. There was a borderline significant result between age and the outcome variable (p = 0.05). However, it should be noted that these factors only predicted a small proportion of the variation not accounted for by the other variables. Interactions between age and childhood adversity variables were also explored but none were statistically significant.

**Table 4:** Results of a univariate GLM for the childhood adversity variables plus age on intended reproductive timing.

					95% Confidence Interval for β	
	F	Sig	ηp^2 1^	β	Lower Bound	Upper bound
Intercept	25.24	0.00	0.08	12.49	6.44	18.53
Age	3.79	0.05	0.01	0.03	0.00	0.05
Neighbourhood Deprivation	5.36	0.02	0.02	0.00	0.00	0.00
Residential Instability	4.95	0.03	0.02	−0.22	−0.41	−0.03
Timing of Father Absence (0 to 14 years)	0.45	0.64	0.00			
Timing of Father Absence (0 to 5 years)				1.91	−0.91	4.73
Timing of Father Absence (6 to 14 years)				0.85	−0.53	2.24
Step-Father Presence	0.02	0.90	0.00	0.94	−1.47	3.35
Biological Brothers	0.34	0.56	0.00	−0.17	−0.75	0.41
Biological Sisters	1.01	0.32	0.00	−0.28	−0.83	0.27
Half/Step Brothers	4.47	0.04	0.02	−0.60	−1.16	−0.04
Half/Step Sisters	0.37	0.54	0.00	0.20	−0.44	0.83
Family Support	4.28	0.04	0.01	0.09	0.00	0.18
Perceived Neighbourhood Safety and Quality	9.90	0.00	0.03	0.18	0.07	0.29

ηp^2 1^: Partial eta squared. This is the proportion of variation not accounted for by other variables captured by the named variable.

### Childhood Adversity and Interest in Infants

We used a multivariate GLM with the seven measures of interest in infants as the outcome variables and childhood adversity factors plus age as the predictor variables. As seen in Table 5, there was only a significant effect of family support on interest in infants such that feeling more supported increased participants interest in infants F(7, 301) = 2.64, p = 0.01. Specifically, higher family support was related to an increased interest in animal infant photos F(1,307) = 4.36, p = 0.04, human infant photos F(1,307) = 5.89, p = 0.02 and human infant silhouettes F(1,307) = 6.24, p = 0.01. There was also a borderline significant effect of CPTT Accuracy such that increased feelings of family support was related to better accuracy at remembering adult faces in the CPTT, F(1, 307) = 3.88, p = 0.05). When interactions between age and each of the independent variables were added, only age and biological brothers was statistically significant, F(7, 291) = 2.99, p = .005. The negative parameter value of the biological brothers variable indicated that as participants get older the effect of brothers on interest in infants becomes weaker.

**Table 5:** Results of a multivariate GLM for the childhood adversity variables plus age on the set of interest in infants measures.

	F	Sig	ηp^2 1^
Intercept	7.33	0.00	0.15
Age	1.06	0.39	0.02
Neighbourhood Deprivation	1.40	0.21	0.03
Residential Moves	1.50	0.17	0.03
Timing of Father Absence	1.32	0.19	0.03
Step-Father Presence	0.39	0.91	0.01
Biological Brothers	1.12	0.35	0.03
Biological Sisters	0.72	0.65	0.02
Half/Step Brothers	0.48	0.85	0.01
Half/Step Sisters	1.22	0.29	0.03
Family Support	2.64	0.01	0.06
Perceived Neighbourhood Safety and Quality	0.82	0.57	0.02

ηp^2 1^: Partial eta squared. This is the proportion of variation not accounted for by other variables that is captured by the named variable.

## Discussion

We measured interest in infants, childhood adversity and intended reproductive timing in a large sample of English adolescent girls. We used several different measures of interest in infants, including a self-report rating item, a previously used preference task, and a novel implicit attentional computer-based measure. We found that these different measures were at best very weakly correlated with one another, highlighting the complexity of the interest in infants construct and the possibility that different measures may not be capturing the same thing. Moreover, none of the interest in infants measures were significantly associated with intended reproductive timing. We found that greater childhood adversity was associated with earlier intended reproduction. However, the control variable (age) was borderline significant suggesting that it is also a factor in intended reproductive timing. We also found that greater family support was a significant predictor of increased interest in infants.

In the introduction, we discussed the hypothesis that interest in infants might be a psychological mechanism activated early in girls who have experienced childhood adversity as a component of their accelerated life-history schedules [20]. This hypothesis predicts that interest in infants should be increased by childhood adversity, and greater interest in infants should be associated with earlier intended reproduction. Neither of these predictions was met in our study. We confirmed previous findings that deprivation, residential instability, more half/step brothers, less family support and poorer perceptions of neighbourhood are all associated with an intention to reproduce younger [9], [13]–[15], [47], [48]. However, not only was the intention to reproduce younger not associated with interest in infants, but the one significant predictor, family support, was in the opposite direction to the prediction of the hypothesis. That is, greater family support, which we also found in a previous study in the same population to be associated with desire for *later* reproduction [15], was here associated with *increased* interest in infants.

Our results thus differ somewhat from those of Maestripieri et al. [20], who found that father absence reduced age at menarche and increased interest in infants, and that there was a weak direct association between interest in infants and markers of reproductive timing. However, similar to our findings Maestripieri et al. [20] did find increased preference to infant stimuli amongst father present girls with more positive family experiences. Still, the reason for the discrepancy with the results of Maestripieri et al. [20] is not clear, since our set of interest in infants measures included the PT that they used, our sample size was much larger and our set of childhood adversity measures more comprehensive. We should note however that in our sample correlations between father absence, step-father presence and intended reproductive timing, whilst in the expected directions, were not significant. These findings were contrary to a large body of previous literature in which father absence has been found to be a predictor of early reproduction [8], [49].

Because we used stated age at first birth as a proxy for potential future reproductive behaviour caution is advised when interpreting the relationships we found between intended reproductive timing and childhood adversity. Although Nettle, Coall and Dickins [9], found stated intentions to be an accurate indication of future reproductive behaviour in a cohort of young British women their sample was in late adolescence at the time of response where as our sample was in early adolescence. Time perspective is thought to be weak during late childhood and early adolescence [50]. We found that older girls in our sample tended to state an older desired age at parenthood. However, age was controlled in all analyses. Moreover our participant's responses were not at all implausible, ranging from 14 to 36 years with a mean of age 25 years.

There is evidence to suggest that reproductive timing is partly heritable [51]. However, childhood adversity tends to be intergenerational making it unclear to what extent the mother-daughter relationship in reproductive timing is the result of genetics or environment. The young age of our participants meant that asking about mother's age at first reproduction might not have produced reliable responses. As such we decided not to include that measure in our questionnaire.

The weak correlations both between and within the interest in infants measures highlight that, despite the long-established literature on this topic, measuring the construct is actually a complex task. It raises the question, what is ‘interest’? An emerging distinction in the literature [40], [43], [52] based on the neural classification of reward [53], suggests that interest in infants could be comprised of the motivation-centred *‘wanting’* and the conscious pleasure-centred *liking*. We attempted to isolate and measure these two facets of reward using the CPTT and the PT, respectively, but it is not clear whether our measures successfully captured this distinction. Ultimately, interest in infants as an adaptation for learning mothering skills should at the very least require ‘interest’ to be an attraction to infant stimuli regardless of how that attraction is operationalised. As Buss [54] argued, albeit while discussing theories of sexual strategies, ‘psychological preferences could not have evolved unless they have consequences for actual behaviour’. Interest merely needs to be sufficient at motivating the individual to interact with an infant thus increasing the chances of acquiring caretaking skills. However, until a consensus method for measuring interest in infants is found it will be difficult to compare across study results.

One possibility for future research could be to modify the PT by replacing the infant versus adult forced choice element with a neutral object versus infant/adult forced choice element. Currently it is unclear whether the PT measures interest in infants or just dislike of the alternative adult images, an issue Maestripieri et al. [20] have addressed. Additionally, with some modification the CPTT has potential to be a reliable tool for measuring interest in infants in early adolescents. Findings in this sample and two other samples have shown that accuracy is better for adult faces compared to infant faces despite participants taking longer to count the purple triangles during the infant trials. This suggests not only that adult faces could be easier to recognise, likely because of their distinctive features, but also that infant faces appear to be more distracting. Therefore the CPTT could be more informative when focusing on the timing rather than the accuracy variable. As well, because of the possible importance of interest in infants in acquiring caretaking skills it might be useful to manipulate emotional salience. Mothers have been found to be particularly sensitive to this manipulation [55], [56].

Our findings suggest that although early childhood adversity speeds up reproductive timing, it does not at the same time increase interest in infants. On the contrary, experiencing greater feelings of family support is indicative of displaying more interest in babies but not necessarily wanting them sooner. In hindsight, this perhaps makes intuitive sense if we consider variations in reproductive strategies. In their seminal paper, Belsky, Steinberg and Draper [1] theorised that children growing up in supportive early environments should go on to invest more in their offspring because such environments are conducive to an individual's growth and development. Adverse early environments, on the other hand, induce a strategy of early attempts at reproduction with relatively little parental investment in each child. Measures of interest in infants may capture something about intended parental investment in offspring, rather than intended timing of reproduction. This is potentially important because women who become mothers young, despite often reporting a desire for early motherhood [9] are also statistically more likely not to breast-feed [57], and to experience post-natal depression, causing a disengagement from their babies [58].

## Supporting Information

Data S1 This is a.csv file that contains all the variables and associated data used in the analysis of this paper.
(CSV)

Table S1 This table gives the name, variable type and description of all variables analysed in this paper.
(DOCX)
