# Supplementary material for: Childhood Adversity Accelerates Intended Reproductive Timing in Adolescent Girls without Increasing Interest in Infants
Source: PLoS One. 2014 Jan 16;9(1):e85013. doi: 10.1371/journal.pone.0085013 (PMC3894174; doi:10.1371/journal.pone.0085013)
Supplement: Table S1 — This table gives the name, variable type and description of all variables analysed in this paper. (DOCX) [file pone.0085013.s002.docx]

Table S1. The name, variable type and description of all variables analysed in the paper.

| Variable | Type | Description |
| --- | --- | --- |
| ID | Nominal | Participants ID number |
| Age_Months | Continuous | Age of the participants in months at time of data collection. |
| Neighbourhood_Dep | Continuous | This is an index of multiple deprivation of small areas in England and Wales. The scores are ranked from 1 (most deprived area) to 32 482 (least deprived). Participant’s IMD score was identified via their postcode. |
| Relocations | Continuous | The participant’s total number of residential relocations. |
| Mother_Absence | Categorical | Mother no longer lives in the same house as the participant. 0=no (mother is present) 1=yes (mother is absent). |
| Father_Absence | Categorical | Father no longer lives in the same house as the participant. 0=no (father is present) 1=yes (father is absent). |
| Age_at_Mother_Absence | Continuous | Age of participant when mother stopped living in the same house. |
| Age_at_Father_Absence | Continuous | Age of participant when father stopped living in the same house. |
| StepFatherPres | Categorical | Step-Father lives in the same house as the participant. 0= no (step father does not co-reside in same house) 1= yes (step-father does co-resides in same house). |
| Timing_of_Father_Absence | Categorical | The time period in the participant’s life when the father stopped living in the same house. 1= 0-5 years, 2= 6 -14years, 3= father is present. |
| Biological_Brother | Continuous | Total number of biological brothers. |
| Biological_Sister | Continuous | Total number of biological sisters. |
| Half/Step_Brother | Continuous | Total number of half/step-brothers. |
| Half/Step_Sister | Continuous | Total number of half/step-sisters |
| Become_Parent | Categorical | Participants reported if they would like to have children one day.0=No, 1=Yes |
| Ideal_Age_Parenthood | Continuous | Participants reported how old they would like to be when they have their first child. Only participants answering ‘Yes’ to the ‘BecomeParent’ variable were asked to answer this question. |
| Family_Support | Continuous | This was a measure of participant feelings of family support. There were five questions measured on a scale from one to seven. The responses were summed with higher scores indicating high feelings of family support. Minimum possible score= 5. Maximum possible score= 35. |
| Perceived_Neighbourhood_Safety_Quality | Continuous | This was a measure of participant feelings of neighbourhood safety and quality. There were eight questions measured on a scale from one to four. The responses were summed with higher scores indicating better perceptions of neighbourhood safety and quality. Minimum possible score=8. Maximum possible score= 32. |
| Fondness_For_Babies | Continuous | This was a self-report measure of how much the participant liked babies. It was measured on a seven point scale where 1= not at all to 7= very much. |
| PT_Animal_Baby_Silhouette | Continuous | This was from the Preference Task. It measured how often the participant chose the baby instead of the adult stimuli as the preferred image. The minimum number was zero and the maximum was five. |
| PT_Human_Baby_Silhouette | Continuous | This was from the Preference Task. It measured how often the participant chose the baby instead of the adult stimuli as the preferred image. The minimum number was zero and the maximum was five. |
| PT_Animal_Baby_Photo | Continuous | This was from the Preference Task. It measured how often the participant chose the baby instead of the adult stimuli as the preferred image. The minimum number was zero and the maximum was five. |
| PT_Human_Baby_Photo | Continuous | This was from the Preference Task. It measured how often the participant chose the baby instead of the adult stimuli as the preferred image. The minimum number was zero and the maximum was five. |
| CPTT_Adult_Kappa | Continuous | This was from the Count the Purple Triangles Task. This is the participant’s average Cohen’s kappa score during the adult trials of the recognition portion of the task. The Cohen’s kappa is the agreement between the participant’s response in the recognition portion of the task and the actual presence or absence of the image. A Cohen’s kappa score of 1 indicates perfect agreement. |
| CPTT_Baby_Kappa | Continuous | This was from the Count the Purple Triangles Task. This is the participant’s average Cohen’s kappa score during the baby trials of the recognition portion of the task. The Cohen’s kappa is the agreement between the participant’s response in the recognition portion of the task and the actual presence or absence of the image. A Cohen’s kappa score of 1 indicates perfect agreement. |
| CPTTAcc | Continuous | This was the difference between the participant’s average Cohen’s Kappa scores (CPTTAcc= CPTT_Baby_Kappa- CPTT_Adult_Kappa) |
| CPTT_Adult_Time | Continuous | This was from the Count the Purple Triangles Task. This is the participant’s average time spent counting purple triangles during the adult trials (in milliseconds). |
| CPTT_Baby_Time | Continuous | This was from the Count the Purple Triangles Task. This is the participant’s average time spent counting purple triangles during the baby trials (in milliseconds). |
| CPTTTime | Continuous | This was the difference between the participant’s average time spent counting purple triangles during the adult and baby trials (CPTTTime= CPTT_Baby_Time-CPTT_Adult-Time) |
